# Supplementary material for: Ecological Risks of Antibiotics in Urban Wetlands on the Qinghai-Tibet Plateau, China
Source: Int J Environ Res Public Health. 2023 Jan 18;20(3):1735. doi: 10.3390/ijerph20031735 (PMC9914113; doi:10.3390/ijerph20031735)
Supplement: Supplementary file 1 [file ijerph-20-01735-s001.zip › ijerph-2136330-supplementary.pdf]

Table S1. Antibiotic standards

Group name	Antibiotics	Brands
Macrolides (MLs)	Erythromycin (ERM)	Dr.Ehrenstorfer
	Roxithromycin (RTM)	Dr.Ehrenstorfer
	Azithromycin (AZM)	Dr.Ehrenstorfer
	Tylosin (TYL)	Dr.Ehrenstorfer
Quinolones (QNs)	Ofloxacin (OFX)	Dr.Ehrenstorfer
	Norfloxacin (NFX)	Dr.Ehrenstorfer
	Ciprofloxacin (CFX)	Dr.Ehrenstorfer
	Enrofloxacin (EFX)	Dr.Ehrenstorfer
	Lomefloxacin (LFX)	ANPEL
	Fleroxacin (FLX)	Dr.Ehrenstorfer
	Difloxacin (DFX)	ANPEL
Tetracyclines (TCs)	Doxycycline (DOX)	Dr.Ehrenstorfer
	Tetracycline (TC)	Dr.Ehrenstorfer
	Oxytetracycline (OTC)	China Inspection Institute
	Chlortetracycline (CTC)	Dr.Ehrenstorfer

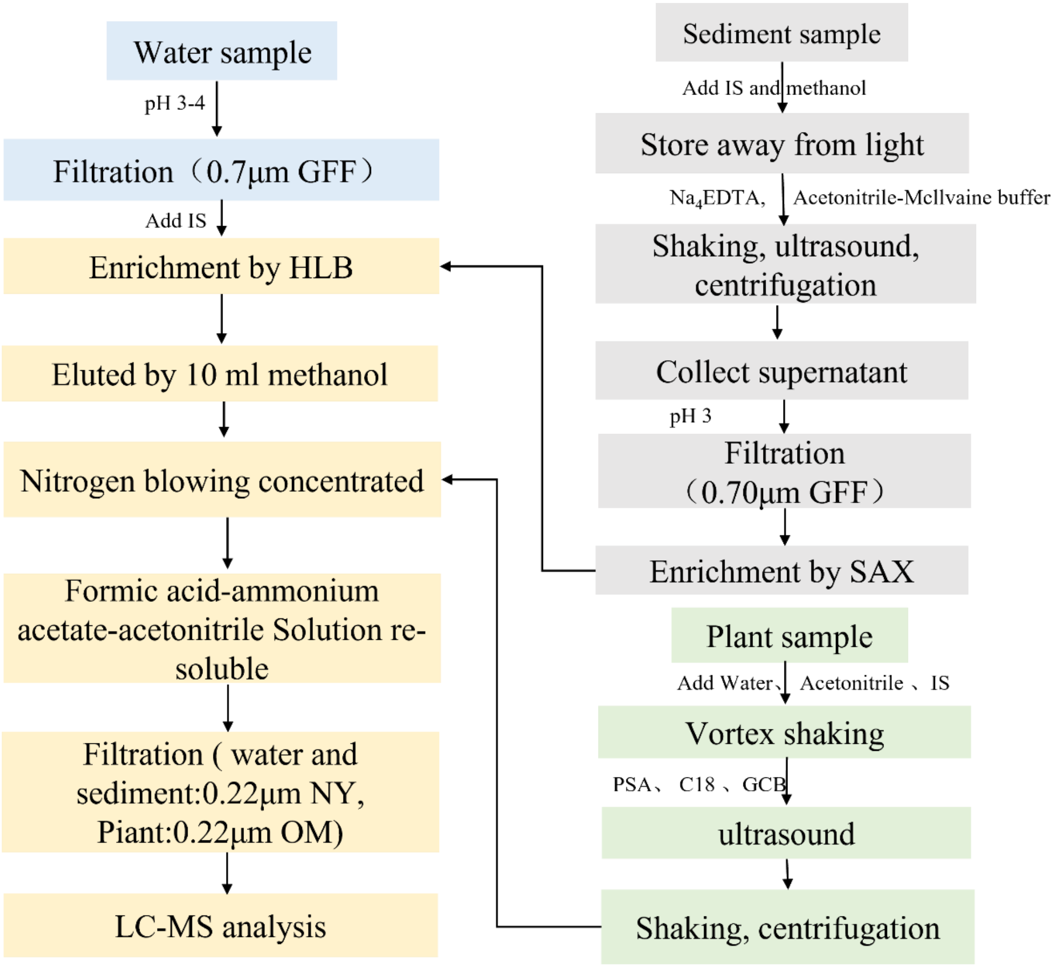

Figure S1. Schematic diagram of the water, sediment, and plant samples preparation procedure

Table S2. Gradient elution step

t/min	Mobile phase A (volume fraction) %	Mobile phase B (volume fraction) %
0.0	90	10
5.0	85	15
7.0	80	20
11.0	89	11
15.0	40	60

Table S3. Conditions for monitoring selective responses to antibiotic protonation or deprotonation in soil and water（$[M+H]^+$or$[M-H]^-$）

Group		Parent ion (m/z)	Daughter ion 1（m/z）	Daughter ion 2（m/z）
MLs	ERM	734.3	576.3	158.0
	RTM	837.4	158.2	679.4
	AZM	749.6	591.4	116.3
	TYL	916.5	174.1	772.3
QNs	OFX	362.3	318.1	261.3
	NFX	320.2	302.12	276.
	CFX	332.1	288.2	245.1
	EFX	360.2	316.2	245.2
	LFX	352.0	265.2	308.1
	NFX	370.3	326.2	269.1
	DFX	400.2	356.3	299.2
TCs	DOX	455.1	428.1	154.2
	TC	455.2	410.3	426.9
	OTC	461.1	426.4	443.3
	CTC	479.3	444.2	462.1

Table S4. Conditions for monitoring selective responses to antibiotic protonation or deprotonation in the plant（$[M+H]^+$or$[M-H]^-$）

Group		Parent ion (m/z)	Daughter ion 1（m/z）	Daughter ion 2（m/z）
MLs	ERM	734.4	158.1	576.3
	RTM	837.5	158.0	679.4

	AZM	749.9	157.8	591.4
	TYL	916.5	174.1	101.1
	OFX	362.1	261.1	318.1
	NFX	320.1	233.0	276.1
	CFX	332.1	314.1	288.1
QNs	EFX	360.2	316.1	245.0
	LFX	352.1	265.1	308.1
	NFX	370.1	269.1	326.1
	DFX	400.2	356.1	299.0
	DOX	455.2	428.2	154.0
TCs	TC	455.3	154.0	410.2
	OTC	461.2	426.2	443.1
	CTC	479.3	444.2	462.2

Table S5. Rotation composition matrix of surface water in the wetland.

Antibiotics	component			
	1	2	3	4
EFX	0.009	-0.118	0.155	**0.656**
CFX	-0.072	-0.066	0.072	**0.640**
OFX	**0.930**	0.049	-0.023	-0.155
NFX	**0.900**	-0.023	-0.065	-0.220
LFX	0.652	-0.270	-0.551	-0.091

FLX	0.195	0.215	-0.858	-0.171
DFX	0.115	**0.958**	0.071	-0.007
OTC	0.836	0.053	0.038	0.151
TC	0.800	0.328	0.038	-0.084
CTC	0.246	**0.931**	-0.051	-0.146
RTM	-0.058	**0.968**	-0.081	-0.049
AZM	0.216	0.175	**0.886**	0.156
ERM	0.731	0.121	-0.017	0.159
TYL	-0.054	-0.224	0.608	-0.600

Extraction method: Principal component analysis.

Rotation method: Varimax with Kaiser normalization.

Table S6. Toxicological data of antibiotics for the most aquatic sensitive species

Analytes	Tested species	Assessment factor	PNEC / (ng / L)	Ref
AZM	*Daphnia sp.*	1000	12000	[1]
TYL	*M. aeruginosa*	1000	34	[2]
NFX	*Vibrio fischeri*	100	103.8	[3]
OTC	*M. aeruginosa*	1000	207	[4]
RTM	*P. subcapitata*	100	100	[5]
OFX	*Vibrio fischeri*	100	11.3	[3]
LFX	*M. aeruginosa*	1000	186	[6]
TC	*M. aeruginosa*	1000	90	[2]
CTC	*M. aeruginosa*	1000	50	
EFX	*V. fischeri*	100	28.8	[3]
CFX	*M. aeruginosa*	1000	5	[2]
DOX	*L. gibba*	1 000	316	[7]
ERM	*C. vacuolata*	1000	6000	[8]
FLX	-	1000	24	[9]

DFX	-	1000	240000	[10]

Table S7. Toxicological data of Ofloxacin (OFX) for the most aquatic sensitive species

Analytes	Tested species	Species group	EC (ng / L)	RQ	Ref
OFX	*Lemna gibba*	Duckweed	532000	2.294849624	[11]
	Cyclotella meneghiniana	Diatom	90600	13.47527594	
	Pseudokirchneriella subcapitata	Algae	4740	257.5654008	
	Pseudomonas putida	Bacteria	10000	122.086	
	Synechococcus leopolensis	Algae	16000	76.30375	
	Vibrio fischeri	Bacteria	90	13565.11111	
	Brachionus calyciflorus	Rotifera	530000	2.303509434	[12]
	Pseudomonas putida	Bacteria	10000	122.086	[13]
	Lemna minor	Duckweed	126000	9.689365079	[6]
	Microcystis aeruginosa	Cyanobacteria	21000	58.13619048	
	Pseudokirchneriella subcapitata	Algae	1440000	0.847819444	[12]
	Ceriodaphnia dubia	Crustacean	26700000	0.045725094	[14]
	Daphnia magna	Crustacean	76580000	0.015942283	
	Ceriodaphnia dubia	Crustacean	17410000	0.070124067	
	Ceriodaphnia dubia	Crustacean	3130000	0.390051118	[12]
	Daphnia magna Straus	Crustacean	31750000	0.038452283	
	Pseudokirchneriella subcapitata	Algae	12100000	0.100897521	[6]

Table S8. BCF of aquatic plant antibiotics in wetlands (L / kg)

Projects	Haihu wetland	Huangshui wetland	Huoshaogou wetland	Beichuan wetland	Ninghu wetland
EFX	426.38	333.17	343.15	530.18	336.07
CFX	109.52	0.00	0.00	0.00	0.00
OFX	3691.47	124.60	1924.93	0.00	1110.87
NFX	1488.74	257.08	1121.43	1978.14	673.05
LFX	776.75	221.39	808.09	619.49	3295.11
FLX	2649.42	0.00	288.42	308.17	2785.60
DFX	479.29	431.81	419.51	419.64	607.52

OTC	79.55	0.00	0.00	0.00	1456.73
TC	0.00	0.00	0.00	0.00	176.88
CTC	0.00	0.00	0.00	0.00	0.00
DOX	0.00	0.00	0.00	0.00	0.00
RTM	0.00	0.00	0.00	0.00	0.00
AZM	415.22	397.88	396.06	200.98	446.60
ERM	676.31	0.00	0.00	0.00	0.00
TYL	3348.26	6659.18	1350.66	1113.49	1094.10

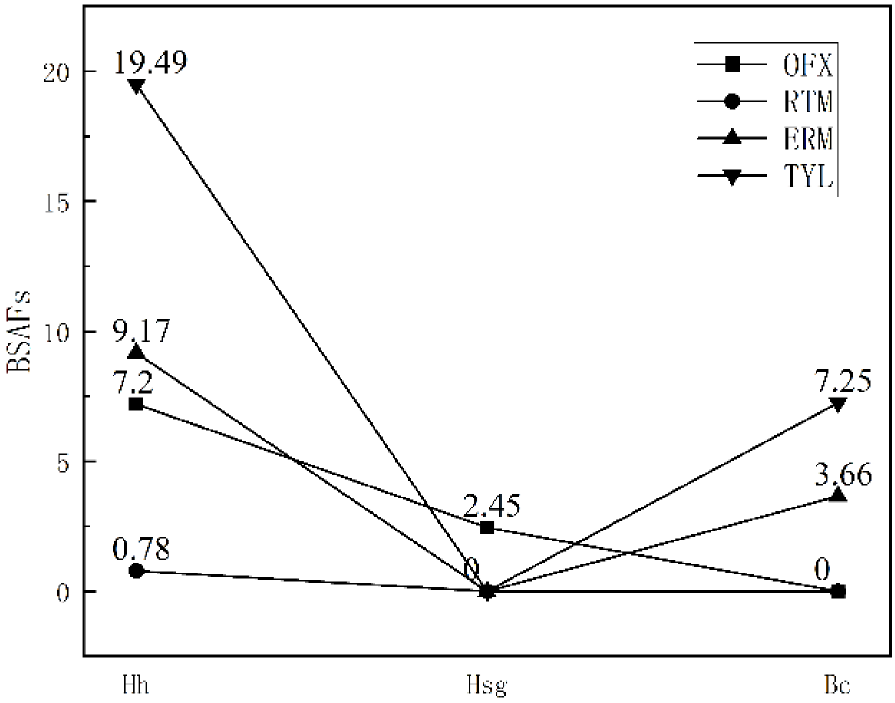

Figure S2. Biota-sediment accumulation factors (BSAFs), Haihu wetland (Hh), Huoshaogou wetland (Hsg), Beichuan wetland (Bc).

References

1. Xue, B.; Zhang, R.; Wang, Y.; Liu, X.; Li, J.; Zhang, G. Antibiotic Contamination in a Typical Developing City in South China: Occurrence and Ecological Risks in the Yongjiang River Impacted by Tributary Discharge and Anthropogenic Activities. Ecotoxicol. Environ. Saf. 2013, 92, 229–236.
2. Halling-Sørensen, B. Algal Toxicity of Antibacterial Agents Used in Intensive Farming. Chemosphere 2000, 40, 731–739.
3. Backhaus, T.; Scholze, M.; Grimme, L.H. The Single Substance and Mixture Toxicity of Quinolones to the Bioluminescent Bacterium Vibrio Fischeri. Aquat. Toxicol. 2000, 49, 49–61.
4. Lützhøft, H.-C.H.; Halling-Sørensen, B.; Jørgensen, S.E. Algal Toxicity of Antibacterial Agents Applied in Danish Fish Farming. Arch. Environ. Contam. Toxicol. 1999, 36, 1–6.
5. Yang, L.-H.; Ying, G.-G.; Su, H.-C.; Stauber, J.L.; Adams, M.S.; Binet, M.T. Growth-inhibiting effects of 12 antibacterial agents and their mixtures on the freshwater microalga pseudokirchneriella subcapitata. Environ. Toxicol. Chem. 2008, 27, 1201.
6. Robinson, A.A.; Belden, J.B.; Lydy, M.J. Toxicity of fluoroquinolone antibiotics to aquatic organisms. Environ. Toxicol. Chem. 2005, 24, 423.
7. Białk-Bielińska, A.; Stolte, S.; Arning, J.; Uebers, U.; Böschen, A.; Stepnowski, P.; Matzke, M. Ecotoxicity Evaluation of Selected Sulfonamides. Chemosphere 2011, 85, 928–933.
8. Ding, H.-J.; Zhong, J.-Y.; Wu, Y.-X.; Zhang, W.-H.; Zou, B.-C.; Lou, Q.; Yang, P.; Fang, Y.-Y. Characteristics and ecological risk assessment of antibiotics in five city lakes in Nanchang City，Lake Poyang Catchment. J. Lake Sci. 2017, 29, 848－858.
9. Silva, L.J.G.; Pereira, A.M.P.T.; Meisel, L.M.; Lino, C.M.; Pena, A. Reviewing the Serotonin Reuptake Inhibitors (SSRIs) Footprint in the Aquatic Biota: Uptake, Bioaccumulation and Ecotoxicology. Environ. Pollut. 2015, 197, 127–143.
10. Ding, J.-N.; Liu, S.-J.; Zou, J.-M.; Shi, J.-Z.; Zou, H.; Shi, H.-X. Spatiotemporal Distributions and Ecological Risk Assessments of Typical Antibiotics in Surface Water of Taihu Lake. Environ. Sci. 2021, 42, 1811–1819.
11. Brain, R.A.; Johnson, D.J.; Richards, S.M.; Sanderson, H.; Sibley, P.K.; Solomon, K.R. Effects of 25 pharmaceutical compounds to lemna gibba using a seven-day static-renewal test. Environ. Toxicol. Chem. 2004, 23, 371.
12. Isidori, M.; Lavorgna, M.; Nardelli, A.; Pascarella, L.; Parrella, A. Toxic and Genotoxic Evaluation of Six Antibiotics on Non-Target Organisms. Sci. Total Environ. 2005, 346, 87–98.
13. Kümmerer, K.; Al-Ahmad, A.; Mersch-Sundermann, V. Biodegradability of Some Antibiotics, Elimination of the Genotoxicity and Affection of Wastewater Bacteria in a Simple Test. Chemosphere 2000, 40, 701–710.
14. Ferrari, B.; Mons, R.; Vollat, B.; Fraysse, B.; Paxéus, N.; Lo Giudice, R.; Pollio, A.; Garric, J. Environmental risk assessment of six human pharmaceuticals: Are the current environmental risk assessment procedures sufficient for the protection of the aquatic environment? Environ. Toxicol. Chem. 2004, 23, 1344.
